# Supplementary material for: Are carbon emissions trading and green financial instruments synergistic? -Comprehensive quantitative research based on content analysis
Source: PLoS One. 2024 Mar 7;19(3):e0298601. doi: 10.1371/journal.pone.0298601 (PMC10919601; doi:10.1371/journal.pone.0298601)
Supplement: S1 Data — (ZIP) [file pone.0298601.s001.zip › Data/地级市Z_F工作报告.zip!/╕ú╜¿/╒─╓▌╩╨/└┤╫╘╣1⁄2╓┌║┼í╛┬φ┐╦╩2╛▌═°í┐_2014.docx]

各位代表：

　　现在，我代表漳州市人民政府向大会作政府工作报告，请予审议，并请市政协各位委员和其他列席的同志提出意见。

　　一、2013年工作回顾

　　过去的一年，在省委、省政府和市委的正确领导下，在市人大和市政协的监督支持下，我市各级政府认真贯彻党的十八大及十八届二中、三中全会和习近平总书记系列重要讲话精神，按照市委的部署，全力推进工业化提速、城镇化提升、现代农业提效，经济社会发展稳中有进、稳中向好，市第十五届人大二次会议确定的各项任务全部完成。

　　全市生产总值2236亿元，增长11.5%；全社会固定资产投资1761.5亿元，增长18.5%；公共财政总收入237.8亿元，增长15.7%，其中，地方公共财政收入154.9亿元，增长17.6%；规模工业增加值897.8亿元，增长15.6%；外贸出口71.08亿美元，增长7.9%；实际利用外资9.46亿美元，增长6.2%；社会消费品零售总额701.3亿元，增长12.9%；城镇居民人均可支配收入26471元，增长10.5%；农村居民人均可支配收入11639元，增长12.0%；城镇登记失业率1.99%；人口自然增长率8.08％。；节能减排“十二五”中期考核任务基本完成。

　　一年来的主要工作和成效是：

　　（一）积极调结构促转型，产业升级步伐加快。三次产业结构由15.9∶47.8∶36.3调整为15.5∶48.8∶35.7。一产巩固提升。新增国家农业产业化示范基地1个、国家级农业标准化示范区2个、省级农业标准化示范区2个、设施农业8.9万亩、无公害农产品60个、绿色和有机产品10个、地理标志商标11个。省级农民创业园、示范基地覆盖所有县（市、区）。漳州被授予“中国石斑鱼之都”，诏安荣获“中国海峡硒都”称号，东山成为全国唯一出口海捕水产品质量安全示范区。海峡花卉集散中心建成投入运营，“海峡两岸新型农民交流培训基地”正式挂牌运作。完成植树造林48.8万亩、水土流失治理40.8万亩。水利投资24.8亿元，长泰枋洋水利枢纽、古雷区域引水工程等17个重大水利项目加快建设。二产提质提效。工业“千百工程”项目完成投资735亿元，增长16.7%；技改投资330亿元，增长29.9%。新增规模工业企业143家，位居全省第1。新认定高新技术企业36家，高新技术企业产值830亿元。漳州国家高新区正式获国务院批准，获得“全国科技进步先进市”四连冠，入选“中欧智慧城市合作中方试点城市”，成功举办首届海峡两岸工业设计科技创新大赛、首届中国钟表设计大赛。钟表基地入选国家外贸转型升级示范基地，金都海洋生物产业园被认定为“国家科技兴海产业示范基地”。海洋产业增加值489.5亿元，增长18.3%。三产发展提速。海峡两岸农产品物流城投入使用。“水仙花乡”网站平台上线运行。漳州名优产品（昆明）展洽会、漳州味（福州）食品展销与订货会等活动成功举办，产品市场进一步拓展。新增限额以上商贸流通企业115家。旅游总收入165亿元，增长16%，新增4A级景区1家。漳浦获评“国家优秀旅游县”，南靖入选“中国旅游竞争力百强县”，云霄获评“中国温泉之乡”。

　　（二）突出抓竞赛打战役，发展后劲明显增强。“五大战役”、“十项竞赛”均超额完成年度目标。重大项目建设方面。全市在建投资超百亿元项目5个、超十亿元项目104个。福欣特钢一期和联盛纸业第7条生产线正式投产，旗滨玻璃第7、8条生产线点火。古雷石化启动项目即将投产，古雷新港城首期安置房建成交付，总投资150亿元的滨海城当年签约当年开工。古雷1600万吨炼化一体化、漳州LNG、漳州核电获准开展前期工作。基础设施方面。交通基础设施完成投资146.5亿元，增长7.1%。厦深铁路漳州段、厦漳跨海大桥和4条高速公路建成通车；新增高速公路里程204公里、农村水泥路472公里，改造危病桥40座。电力建设完成投资20.1亿元，增长27%。

　　（三）坚持高起点高标准，城乡建设一体推进。《漳州市城市总体规划（2012-2030）》、《漳州（古雷-南太武）滨海新区发展规划》以及13项专项规划编制完成。新改扩建城市道路112公里，新增公园5个、绿道40公里、供水管网21公里、污水管网12公里、垃圾中转站3座。蒲姜岭生活垃圾焚烧发电厂建成点火调试。开展“公共交通建设年”活动，实施交通秩序大整顿，新投放新能源公交车150辆。“点线面”综合整治投入55亿元，完成计划的244%；“创城”活动深入开展，数字化城市管理信息系统启动建设，“大城管”体系投入试运行。长泰获“国家卫生县城”命名。成功举办福建（漳州）美丽乡村博览会，6个村入选农业部“美丽乡村”创建试点村，漳州高效农业型美丽乡村建设被列为全国十大模式之一。

　　（四）立足激活力添动力，改革开放深入推进。重点领域改革加快推进。小城镇综合改革试点工作扎实有效。事业单位分类改革全面启动。农村综合改革深入开展，集体林权制度改革进一步深化，商品林经营、森林综合保险和林权抵押贷款有序推开。国有企业投融资能力明显增强。出台《整合做强市属重点国有企业工作方案》，市属重点国有企业直接融资132.6亿元，增长69.7%。首支政府引导型创业投资基金正式设立。金融支持力度进一步加大。新增分行1家、支行8家、村镇银行1家，全年本外币贷款余额1420.4亿元，增长17.1%；存款余额1851亿元，增长21.3%。“三维”对接成效明显。成功举办深圳和北京民营企业家座谈会、海外侨商投资漳州（上海）推介会，开展漳籍民营企业家大走访、“海外侨商漳州行”等活动，累计列入省级跟踪对接“三维”项目392个，总投资5934.1亿元。全年对接民企项目187个，总投资1298.4亿元，位居全省第2。漳台交流合作更加密切。海峡两岸“农博会·花博会”成效更好，现场销售额6600万元，签订购销订单28.6亿元，落实签约项目72个，总投资313.3亿元。成功举办第六届海峡两岸开漳圣王文化节、第二届创业漳州台商大会、第五届海峡百姓论坛、首届两岸青年创业家园论坛等活动，两次赴台经贸恳亲活动落实16个合同项目，总投资27.8 亿美元。对外交流交往持续扩大，成立APEC商务协会，新增2个国际友城。

　　（五）致力惠民生促和谐，群众生活持续改善。全市财政民生支出195.4亿元，增长18.1%。就业和社会保障水平提高。新增城镇就业5.23万人、转移农村劳动力7.15万人。城乡居民养老保险实现全覆盖。城乡居民基本医疗保险补助标准和农村低保保障标准进一步提高，保障性住房建设超额完成省定任务，城乡低保、医疗救助、抚恤补助、灾害救济、慈善资助等救助体系逐步完善。圆满完成第三批整村推进扶贫开发任务，实施“造福工程”危房改造8800户，搬迁34755人。5个县（市、区）被确认为“原中央苏区县”。社会事业协调发展。开展“医疗卫生事业三年行动计划暨2013年建设年”活动，新增床位2366张、卫技人员1744名。创建10所省级达标高中校、270所义务教育标准化学校。新改扩建45所公办幼儿园，建设校安工程40个、城区学校扩容工程项目24个，新购国标校车106辆。新增博士后科研工作站1个、享受国务院特殊津贴专家2名、高级职称人才2207人。芗剧《保婴记》获第十三届中国戏剧节优秀剧目奖、导演奖和表演奖，首届国际公共艺术展成功举办。圆满完成“文化宝岛校园行”活动。漳浦被命名为“中国书法之乡”，3个村列入中国传统村落名录。市档案馆晋升为国家一级档案馆。第十二届市运会圆满举行，省运会筹备工作进展顺利，群众体育工作居全国先进行列。防灾减灾能力提升。有效应对“西马仑”、“天兔”等强台风和暴雨袭击。制定实施《防震减灾工作纲要》，深入开展城乡建筑物抗震能力普查，粮食应急供应网点覆盖所有乡镇。生态市建设持续推进。全面开展PM2.5监控和治理，主要水系Ⅲ类以上水质比例占94.4%，空气质量全部达到或优于国家二级标准。漳州生态城市竞争力位居全国第21、全省第1。南靖、长泰获“国家生态县”命名。龙文被确定为“全国国土资源节约集约模范区”，东山入选全国首批国家级海洋生态文明示范区，漳州开发区获评“国家水利风景区”。社会保持安定稳定。推行社会稳定风险评估，推进和谐征迁，推广倾听群众诉求常态化机制和信访听证评议，社会矛盾纠纷排查调处机制进一步完善。深化“平安漳州”建设，完善社会治安防控体系，全面推广社区网格化管理。严格落实安全生产“一岗双责”，深入开展安全整治“三项行动”，推进企业安全标准化建设，安全生产四项控制指标全面下降。军政军民团结巩固发展，荣获“全国双拥模范城”五连冠。食品安全监管体系进一步完善。妇女儿童、青少年、民族宗教、新闻出版、社会科学、行政复议、地方志、科普、气象、残疾人、红十字会、慈善、工会、统计、人防、物价、国防动员和支前双拥等各项工作都取得新的进步。

　　（六）注重转作风提效能，政府自身建设得到加强。严格执行中央“八项规定”，深入开展“四下基层”活动，大力推行“马上就办”，出台《机关工作人员效能问责办法》，组织开展乡镇综合考评。深化行政审批制度改革，持续推进政务服务体系标准化建设，成为全省审批事项最少、流程最简、时限最短的设区市。市行政服务中心荣获三项国家级荣誉。"12345"政务服务热线启动运行。法治政府建设成效明显，依法行政绩效考评位居全省第1。认真执行人大决定决议，自觉接受人大、政协监督，积极配合人大、政协开展检查、视察、调研等活动，全年共办理市人大代表建议178件、市政协提案359件，办结率均为100%。广泛听取各民主党派、工商联、各人民团体和社会各界人士意见建议。在全省设区市中率先全面公开财政预决算和“三公”经费，全市政府部门“三公”经费下降14%。行政监察和审计监督力度加大，廉政建设扎实推进。

　　成绩的取得来之不易，这是省委、省政府和市委正确领导的结果，是全市人民团结拼搏、攻坚克难和各方面大力支持的结果。在此，我谨代表市人民政府向全市人民，向人大代表、政协委员、各民主党派、工商联、各人民团体、无党派人士、离退休老同志和社会各界人士，向中央、省驻漳机构和驻漳部队、武警官兵、公安政法干警，向关心支持漳州发展的港澳台同胞、海外侨胞和国际友人，表示崇高的敬意和诚挚的感谢！

　　在总结成绩的同时，我们也清醒地看到，发展还面临不少困难和问题，主要是：经济总量偏小，产业结构不优，产业龙头企业偏少，产业链延伸不够，自主创新能力不强；城乡公共基础设施配套不足，城市综合管理有待加强，城市面貌有待改观，城区交通不畅和违法占地、违法建设问题仍然突出；城乡、区域发展不协调，基本公共服务不均衡，居民收入持续较快增长难度大，教育、卫生、文化等社会事业还不能满足人民群众的需求；土地、资金、人才、资源环境等要素制约比较突出；征地拆迁、安全生产、社会治安、食品药品安全等方面还存在不少问题；机关服务质量和效能建设有待进一步提升，个别公务人员的担当意识和服务能力不强，消极腐败现象和不正之风不同程度存在。这些问题，我们将高度重视，认真解决。

　　二、做好2014年工作

　　2014年政府工作的总体要求是：深入贯彻党的十八大及十八届二中、三中全会和习近平总书记系列重要讲话精神，全面落实中央和全省经济工作会议、城镇化工作会议、农村工作会议的部署，坚持稳中求进、改革创新，集中开展“项目建设年”、“深化改革年”、“作风建设年”活动，突出产业发展，突出深化改革，突出城乡建设，突出民生改善，突出生态建设，突出作风转变，全力推进富美漳州建设。

　　经济社会发展的主要预期目标是：地区生产总值增长12%，全社会固定资产投资增长23%，规模工业增加值增长18%，公共财政总收入增长15%，其中，地方公共财政收入增长13%，外贸出口和实际利用外资均增长7%，社会消费品零售总额增长14%，城镇登记失业率控制在3.6%以内，人口自然增长率控制在8.8％。以内，城镇居民、农村居民人均可支配收入均增长12%，节能减排指标控制在省政府下达范围之内。

　　为实现上述目标任务，我们要按照尤书记来漳调研时提出的“三次产业协调发展、经济结构优化、产业竞争能力强、城镇化具有特色、生态环境优美、社会安定和谐”的要求，认真贯彻市委十届七次全会精神，紧紧围绕重点领域、重点区域、重点工作，抓项目、扩投资，抓龙头、铸链条，抓发展、惠民生，实施深化“五大战役”重点行动计划，充分发挥消费的基础作用、投资的关键作用、出口的支撑作用。重点抓好七个方面工作：

　　（一）舞活龙头增强产业综合实力

　　突出工业重点，强化产业支撑。实施产业转型升级行动计划和产业龙头促进计划，开工建设十大产业项目。发挥龙头企业作用，大力发展总部经济。开展产业链招商，加快延伸产业链条、做优产业配套，打造4个千亿元级产业集群。继续实施工业“千百工程”，力争完成工业投资900亿元以上。加快引办一批高端化、高质化、高新化临港工业项目，促进海洋经济发展壮大。进一步落实甲供甲控，优先采购本地产品。建立以实际投资和税收贡献为导向的产业促进机制，完善工业增产增效政策，扶持小微企业发展，加快企业异地搬迁、退城入园，加大工业闲置用地厂房清理；把供地数量与建设速度、投资强度、产出效益密切挂钩，鼓励零地扩张、建设多层厂房，促进土地集约节约利用。

　　突破重点区域，促进产业集聚。举全市之力推进古雷开发建设，力促《古雷石化基地发展规划》早日获批，积极申报综合保税区、两岸石化产业深度合作试验区，创造条件设立石油化工自由贸易区；加快启动石化四、五次加工项目，完善基础设施配套，统筹做好居民搬迁安置工作，加快建设古雷石化新港城。加快厦门港南岸区域开发，主动参与厦门东南国际航运中心建设，深化港口资源整合共建，完善港口集疏运体系，力争实现厦门港同港同策。高起点规划厦门港南岸新城，着力打造一港两岸联相呼应、对外更加开放、漳台深度合作、与厦门联动协调发展的新兴生态型港口工贸城市。扶持龙海重振工业雄风，加快推进龙海经济开发区建设。进一步推动台商投资区优化布局、完善配套，承接台湾资金、产业、技术、人才转移。发挥侨优势，支持常山经济开发区规划建设海西滨海旅游华侨城。继续推进各级各类开发区创新体制、加快发展，全力打造11个特色园区。

　　加快三产发展，优化产业结构。规划布局若干个现代服务业集聚区，支持龙文加快现代服务业综合改革试点工作，吸引知名服务企业入驻。加快12个重点物流项目建设，完善制造业供应链服务。实施开拓市场“百千万工程”，加快“电商换市”步伐，培育一批电子商务专业园区，建设“漳州味”电子商务特色平台。进一步整合旅游资源，积极申报“中国温泉之城”，加快东山生态旅游海岛建设，全域规划大乌山旅游景区，加快十大重点旅游项目开发，鼓励发展乡村旅游业。

　　坚持创新驱动，提升产业层级。创新国家级漳州高新区管理体制和运行机制，引导高新技术产业集聚发展，打造新的增长极。实施企业技改项目200个，完成技改投资350亿元以上。加快建设“一园三城四基地五平台”，整合海洋科技创新资源，提升“6·18”科技成果对接转化能力，扎实推进“蓝火计划”，发展科技中介服务，重视引进高级和紧缺专业人才，加速壮大创新人才队伍。

　　（二）夯实基础推动现代农业提质增效

　　稳定发展粮食生产。落实强农惠农富农政策，完成全市174万亩、69万吨粮食生产计划。加快粮食储备现代化仓容建设，支持粮油生产、流通企业发展。加强副食品基地建设，推进农超对接，确保市场供应量足价稳。实施农产品质量提升工程，推广使用有机肥。建立最严格的食品监管制度、原产地可追溯制度和质量标识制度，确保人民群众“舌尖上的安全”。

　　发展壮大特色产业。做强做优“一县一业”，着力打造八大百亿特色产业集群。大力发展农产品精深加工，新增3家产值超十亿元的龙头企业。积极发展林下经济，培育中药材、珍贵家具用材等产业，科学规划开发富硒健康农业资源。继续扶持远洋捕捞和离岸养殖。加快产销市场、信息服务平台建设。整合提升农产品物流市场，引办一批农产品冷链系统、配送中心项目。

　　扶持设施农业发展。制定设施农业发展规划，今年市财政安排1000万元，重点扶持各类普及型设施农业。实施种业创新与产业化工程，推进欧中现代农业技术研发中心、漳台农业种业合作研发中心建设，建立繁育与示范基地20个。加强水利设施、农田基础设施建设，实施22个重大水利项目和170个农村重点水利项目，加快新一代气象雷达项目建设。

　　提高园区建设水平。创新漳浦台湾农民创业园发展模式，推进各类农民创业园、示范基地建设，完善园区基础设施配套。加快龙海、漳浦2个国家级农业产业化示范基地建设，争创国家级农业标准化示范区2个、省级农业标准化示范区4个。提升“农博会·花博会”办会水平，支持办好“世界百合大会”。

　　创新农业经营体系。积极发展家庭农场、专业合作社。健全县、乡、村三级土地流转服务网络，积极探索土地承包经营权信托、入股合作等模式，促进土地有序流转。依托海峡两岸新型农民交流培训基地，大力培养新型农民。深化国有农（林）场改革。发展壮大村集体经济。出台农村金融服务政策，创新农村产权、经营权抵押贷款担保方式，扩大政策性农业保险范围。

　　加快富美乡村建设。开展“一乡镇一示范村”百个富美乡村创建活动，以点带面加快建设一批百姓富裕、生态优美、文化先进的富美乡村。融入闽南古厝文化元素，加强村庄规划建设。深化村庄环境综合整治，着力改善农村人居环境，改造提升农村道路等级，实施饮水安全工程。

　　（三）激发活力全面推进深化改革

　　推进市场准入平等化。落实《整合做强市属重点国有企业工作方案》，支持国有企业更多地参与基础设施、公益项目的投资建设，建立健全“四项制度”，确保国有资产保值增值。落实支持民营经济发展各项政策措施，鼓励民间资本以多种方式进入基础产业、社会事业以及特许经营领域，健全扶持本地民营企业发展和在外漳籍民企回归创业的工作机制，加快民营经济发展壮大。大力发展混合所有制经济，激发各类市场主体活力。

　　推进资源配置市场化。深化土地要素配置机制改革，探索建立存量土地盘活、土地产出效益与新增建设用地计划指标分配挂钩制度，推进工业用地弹性管理制度改革，建立低效利用土地市场化退出机制。建立完善居民用水、用电阶梯价格制度和企业超定额用水加价制度。推行公共服务市场化改革，健全政府购买公共服务机制。整合规范政府采购、国有产权（物权）和工程项目招投标等公共资源交易平台。认真实施第三次全国经济普查。

　　推进融资渠道多元化。深化财政体制改革，增强公共服务能力。加强政府性债务资金投向管理，推进预算绩效管理，建立以绩效为导向的财政专项资金管理机制。继续用好应急周转金、增信资金、助保基金，设立创业投资引导基金，切实帮助中小微企业解决融资难问题。推进“区域集优”直接债务融资工作。加快引银入漳，支持发展村镇银行。加大企业上市扶持力度，鼓励企业进入场外资本市场挂牌，成立海峡股权交易中心漳州运营中心。

　　推进营商环境便利化。稳步推进大部制改革，全面完成市县政府机构改革，稳妥推进事业单位分类改革。健全公务员分类管理制度，深化事业单位人事制度改革。按照“五个统一”要求，推进行政审批规范化、服务标准化。全面推广“123”政务服务模式，努力为企业、群众提供快捷和无差别的服务。开展工商登记制度改革试点，推进工商注册制度便利化。

　　（四）以人为核心推进新型城镇化

　　优化空间布局。加快实施“中心东移、跨江南扩、面海拓展”城市发展战略，制定并实施城镇化发展规划和政策措施，精心编制厦门港南岸新城和古雷石化新港城总体规划。坚持“三规融合”、“四先四后”，增强引导和控制城镇开发建设的能力。加强景观和建筑风貌规划，把燕尾翘脊、红砖红瓦等闽南建筑风格更多体现到城镇规划建设中。加快旧城改造、古城保护和新区拓展，加紧九龙江西溪南岸规划建设，着力推进圆山新区开发，加快形成“一江两岸”的城市发展格局。启动规划建设闽南文化生态博览城，从圆山新区至东南花都，规划建设体现闽南古厝文化特色、绿道串联成线、与花卉苗木产业带相融合的闽南文化生态走廊，努力建成“田园都市、生态之城”的核心区。组织实施《厦漳泉大都市区同城化总体发展规划》，加快厦漳同城大道建设进度，开工建设厦漳城际轨道R3线。

　　完善功能配套。实施城乡基础设施提升行动计划，突出抓好“六项提升工程”，推动城镇基础设施向农村延伸。加大市政设施投入力度，新增和改建城市道路307公里、燃气管网162公里、雨污管网535公里、市政供水管网165公里。加强公交站场建设，新增120辆公交车、1.83万个停车位。开工建设省级1号绿道（滨海绿道）漳州段、市级1号绿道、南江滨湿地公园一期工程和20片150亩城市片林，新建绿道230公里以上。启动“四馆一院”建设，续建人民广场，新建10个城市小广场。

　　提升管理水平。以创建全国文明城市为载体，理顺城镇管理体制，推进“智慧城市”、“数字城管”建设。做好城区4条主要道路夜景照明工程的改造提升，加强市区内河整治，推进市区广告按规划、市场化建设。延伸拓展“点线面”攻坚，启动城区“三边三节点”景观风貌建设，推动城乡环境综合整治全覆盖。开展“六项整治”和“六个一批”评选活动，组织实施“两违”专项整治行动，加强闲置建设用地清理处置，盘活土地存量，加强土地收储。

　　壮大县域经济。完善支持县域经济发展的政策体系和考评激励机制。建立县域协调机制，创新山海协作模式，鼓励发展“飞地经济”。支持各地办好重点工业园区，壮大县域经济实力。深入实施“大城关”发展战略，按照次中心城市标准，加强县城规划建设。加大交通基础设施投入，完成投资150亿元以上。重点实施4个港航项目、5个高速公路项目、44个普通公路项目，加快城际快速通道和县际通道建设，促进县域协调发展。

　　创新体制机制。推进长泰、东山城乡一体化改革试点工作。深化小城镇综合改革建设试点，推进强镇扩权改革，赋予与人口、经济规模相适应的管理权。深化户籍制度改革，全面放开建制镇和县城落户限制，把进城落户农民纳入城镇住房和社会保障体系，推动凭居住证享受与当地居民同等的公共服务和社会福利。以古雷开发区、台商投资区为试点，加快推进农业转移人口市民化，努力破解城乡二元结构。

　　（五）扩大开放拓展对外合作空间

　　多渠道拓展外贸市场。用好促进外贸发展各项扶持政策，努力增强产品市场竞争力。提高通关便利化水平，推广关检“三个一”工作模式，加快无纸化通关改革，推动厦门港“同港同策”和“关检协作”。组织企业赴境外重点市场参展办展，鼓励支持企业设立境外营销网点、开展国际品牌并购。指导企业用好相关自贸区协定、双边多边贸易协定的最新优惠政策，有效应对“两反两保”等各种贸易摩擦。

　　大力度推进“三维”对接。整合招商资源，搭建统一的招商平台，组建专业招商队伍，推行小分队招商。深化项目对接和园区合作，编制产业链招商目录，提高招商引资的针对性和实效性。办好创业漳州台商大会、漳台经贸恳谈会，积极参加第18届中国投洽会、“6·18”海峡项目成果交易会，主动对接大型央企、知名民企、台湾百大企业、重点侨商和世界500强企业，推动招商引资取得新突破。

　　全方位深化漳台合作。发挥“三大平台”作用，建立健全漳台深度合作机制，加快建设对台交流合作示范区。积极争取放宽特殊区域台商投资石化、特殊钢铁产业持股比例等限制。落实ECFA后续协议和《海峡两岸服务贸易协议》中先行先试条款。争取在台设立投资促进机构，加强漳台行业协会对口交流，推动漳台旅游协会互设办事处，探索设立东山台湾食品免税区，积极争取成为赴台个人游试点城市，推进漳台两地客货轮直航。建立漳台文化交流常态化机制，参加第五届开漳圣王文化联谊大会，加快建设一批对台文化交流合作基地，积极承办海峡论坛活动项目，扩大基层、民间交流交往。

　　（六）加大投入切实保障和改善民生

　　着力办好惠民实事。继续集中新增财力，办好20件为民实事项目，开工建设十大市级社会事业重点项目，切实解决关系群众切身利益的突出问题。

　　推进扶贫开发攻坚。落实《关于进一步加强扶贫开发工作的实施意见》，着力推进3个省级扶贫开发重点县、150个贫困村扶贫开发，扶持原中央苏区县和革命老区、少数民族集聚区、库区、海岛及边远山区等欠发达地区加快发展。筹划建设闽粤经济合作区，争取特殊发展政策。实施第四批整村推进扶贫开发，开展结对帮扶和“百龙联百村”、“百名科技人员挂钩百家企业”活动，广泛动员全社会力量参与扶贫攻坚。推进“造福工程”，全年完成危房改造6000户。

　　健全社会保障体系。落实更加积极的就业保障政策，建立健全就业创业保障机制，重视抓好高校毕业生就业工作。完善农村征地补偿标准和多元补偿机制，妥善解决失地农民就业和社会保障问题。健全工资正常增长机制，完善最低工资和工资支付保障、企业工资协商制度。加快金保工程建设，统筹城乡医保管理，提高大病医疗保险报销比例。新开工建设保障性住房8311套，力争建成10500套。积极稳妥推进棚户区、城中村、旧房改造。继续实施城乡低保、农村五保扩面提标工程。出台鼓励社会力量参与社会事业发展优惠政策，大力发展老年服务事业和产业，新建10个农村敬老院。开工建设市社会福利中心。支持发展残疾人事业，保障妇女儿童、青少年合法权益。

　　提高全民健康水平。深化医药卫生体制改革，继续实施医疗卫生事业三年行动计划，全年新增床位2000张、卫技人员1000名，培训全科医生和乡村医生4000名。开展“建设人民满意医院”活动，实施基层中医药服务能力提升工程，巩固和拓展基本药物制度。推进公立医院改革、基层医疗卫生机构改革，吸引社会资本办医。推动村卫生所信息化建设。坚持计划生育基本国策，落实计生新政，治理“三高”问题，提高出生人口素质。

　　促进教育均衡发展。推进教育资金、资源向农村地区、贫困地区、城市新区倾斜，合理划分市区中小学施教区，督促民办学校承担义务教育责任，促进教育均衡发展。实施教学设施配套和优质师资引进工程，支持名校办分校、老校带新校、强校扶弱校，扩大优质教育资源覆盖面。实施新一轮学前教育发展三年行动计划，新开工建设13个公办幼儿园，鼓励有条件的农村小学创办附设幼儿园（班）。全面完成标准化学校建设，新开工建设中小学校安工程6万平方米、城区中小学扩容工程5万平方米。做大做强职业教育，启动建设中职教育园区，支持高职院校扩权提质、加快发展。扶持特殊教育，重视老年教育，发展继续教育、社区教育，构建终身教育体系。加强科普工作，提高全民科学素质。

　　增强文化整体实力。深化文化体制改革，推动经营性文化事业单位转企改制、公益性文化事业单位合并重组，创新公共文化服务向社会购买机制，建立符合文化市场运行的体制机制。开展文化产业（事业）竞赛活动，规划建设文化创意园区。实施文化惠民工程，加快推进市县两级重点文化设施、乡村文化健身场所建设，广泛开展群众性文化活动。建立文艺专业人才奖励机制，留住骨干人才、引进紧缺人才、培养后备人才。加强文化精品创作，组织开展第七届百花文艺奖评选活动，支持以漳州为题材的各类剧作创作。加强历史文化名城名镇名村保护，办好全国“海丝”精品九城市联展。

　　全力办好省运会。加快推进体育场馆和接待酒店建设，确保如期保质交付使用。统筹做好经费预算、器材采购、赛事组织、接待服务等各项工作。加大优秀运动员培养、引进力度，提升竞技体育水平。组织开展“全民健身与省运会同行”活动，促进群众体育和竞技体育同步发展。

　　加强社会治理创新。全面推进“平安漳州”建设，加强基层基础建设和社会治安综合治理，深化社区网格化建设，加大黑恶势力等违法犯罪打击力度。培育发展和规范管理社会服务组织，建立矛盾纠纷排查调处化解综合机制，完善市县联合接访、信访终结等信访工作制度，依法处理信访事项。落实安全生产“四项制度”，推进企业安全生产标准化及安全园区、安全社区创建工作，深入开展重点行业和领域的专项整治，加强古雷石化产业园区安全监管，继续推进道路交通安全综合整治三年行动和市区智能化交通建设。加快公共安全预警防控和应急处置体系建设，重视防震减灾工作，有效应对各种自然灾害和突发公共事件。做好新形势下的外事侨务、民族宗教、双拥优抚工作。

　　（七）发挥优势加强生态文明建设

　　推进生态建设。积极争创国家生态市、生态文明示范区，支持南靖、长泰建设全国生态文明建设试点示范区、东山建设海洋生态文明示范区。突出抓好“一环”（环城市周边）、“三沿”（沿路、沿江、沿海）一重山“青山挂白”整治，坚决遏制乱采滥挖现象。加大养殖面源污染整治，加强“四海”（海湾、海岸、海滩、海水）等自然资源保护，给广大群众、游客和子孙后代留下美丽的海湾、海滩。加快沿海防护林、城市森林、郊野公园、湿地公园、森林公园和生态文化走廊等“六大工程”建设，构筑开敞的绿色空间。加快国家森林城市创建步伐，持续推进“四绿工程”，继续开展“六大森林工程”建设，完成造林绿化14.6万亩。重视湿地保护和湿地生态功能提升，实施山体、水域、海岸带生态修复工程以及工矿废弃地恢复治理，治理水土流失面积15.2万亩。

　　狠抓节能减排。严把项目节能环保关，发展节能环保产业。创新生态保护管理体制，完善生态补偿和资源有偿使用机制。加快重点减排工程建设，推进工业园区污染集中处理，落实重点行业脱硫脱硝工作，加大落后产能淘汰力度。完善污水配套管网，实施一批污水处理扩建工程，提高城镇污水收集处理率。

　　整治薄弱环节。实施“蓝天碧水净土”工程，优先保障饮用水源安全，健全重点流域长效治理机制，推进重点区域畜禽养殖污染整治，加大病死畜禽无害化处理设施投入力度，严厉打击流域违法排污、滥采滥挖等破坏环境行为。加强点源、面源、移动源污染防治，做好六项空气质量新标准指标检测，改善并保持良好的空气质量。实施环保监管能力建设三年行动，加强重金属污染防治和危险废物监管，提高防范环境风险能力。加快推进八尺门退堤还海等海洋生态修复工程，加强海洋环境保护，从严管理用海审批，进一步加强海砂河砂开采管理。

三、加快建设法治政府和服务政府

　　政府工作必须为人民服务、对人民负责、受人民监督、让人民满意，切实增强政府公信力和执行力。

　　依法行政。严格依法办事，认真执行人大决定决议，依法接受人大及其常委会的法律监督和工作监督，认真办理人大代表建议、批评和意见；主动接受人民政协的民主监督，支持人民政协和各民主党派、工商联、无党派人士履行职责，认真办理政协提案；自觉接受社会各界和人民群众的监督。坚持把依法行政的要求贯穿到政府决策、执法、落实的各个环节，加快建设法治政府。

　　高效施政。把心思放在谋发展上，把精力花在干事业上，把功夫用在抓落实上，树立“服务是对外开放第一竞争力”的理念，倡导“马上就办”，像孝顺父母一样做好项目服务工作。简化行政审批，保持审批事项最少、流程最简、时限最短，让企业和群众少跑腿、少受累，甚至足不出户就能办成事。强化效能督查，完善绩效评估，严格行政问责，治理庸懒散奢，推进政务服务提速提质提效。

　　务实理政。政府工作就是要实实在在，脚踩地上。弘扬“弱鸟先飞”、“滴水穿石”、“钉钉子”精神，功成不必在我任，一任接着一任干，一张蓝图绘到底。转文风改会风，少发文、发短文，发管用的文；少开会、开短会，开解决问题的会；少讲话、讲短话，讲群众听得懂、记得住、用得上的话。“一分部署，九分落实”。抓落实办实事，工作闻风而动、雷厉风行；对看准、议定的事，定了就办，办就办好，不拖拖拉拉、不半途而废；工作讲求实际，注重实效，不图虚名，不务虚功，不搞花架子，不做表面文章。

　　廉洁从政。严格执行中央“八项规定”和《党政机关厉行节约反对浪费条例》、《党政机关公务接待管理规定》，严控“三公”经费，让政府过紧日子、人民过好日子。牢记“教训就在身边、诱惑就在眼前、陷阱就在脚下、成败就在手中”，全面落实党风廉政建设责任制，更加重视预防腐败，推进政务公开，把权力和私欲关进制度“笼子”，加大惩治腐败力度，切实做到勤政优政、廉政严政。

　　各位代表，面对新的使命、新的征程，我们责任在心、重任在肩。让我们更加紧密地团结在以习近平同志为总书记的党中央周围，在省委、省政府和市委的坚强领导下，紧紧依靠全市人民，凝心聚力，振奋精神，改革创新，锐意进取，为加快建设富美漳州而努力奋斗！
